# Multimodal Imaging in Extratemporal Epilepsy Surgery

**DOI:** 10.7759/cureus.2338

**Published:** 2018-03-18

**Authors:** Christian Vollmar, Aurelia Peraud, Soheyl Noachtar

**Affiliations:** 1 Epilepsy Center, Dept. of Neurology, University of Munich Hospital, Ludwig-Maximilians-University Munich; 2 Dept. of Neurosurgery, University of Munich Hospital, Ludwig-Maximilians-University Munich

**Keywords:** epilepsy surgery, multimodal imaging, extratemporal epilepsy, image fusion

## Abstract

Neuroimaging is crucial for the evaluation of patients considered for resective epilepsy surgery. Multimodal image fusion is a new tool to integrate all available localizing information on the individual epileptogenic network in a three-dimensional (3D) manner to plan invasive EEG recordings and delineate the epileptogenic zone from the eloquent cortex for the neurosurgical planning of a tailored resection. Here, we illustrate the multimodal fusion of images from different modalities in a patient with medically intractable non-lesional frontal lobe epilepsy who underwent partial frontal lobe resection, rendering him seizure-free.

## Introduction

Epilepsy is one of the most common chronic neurological disorders. Although the majority of epilepsy patients will become seizure-free with currently available antiepileptic medication, about one out of three epilepsy patients will continue to have seizures despite adequate medication. Since etiologies like cortical dysplasia and mesial temporal sclerosis tend not to respond as well to medication, such as in stroke-related epilepsy [1], the option of epilepsy surgery should be evaluated early in the course of epilepsy in these patients. Late surgery is associated with less favorable outcomes [2]. Neuroimaging plays an essential role in the evaluation of patients considered for epilepsy surgery. The currently used techniques include magnetic resonance imaging (MRI), fluorodeoxyglucose positron emission tomography (FDG-PET), and single photon emission computed tomography (SPECT) reflect different aspects of the epileptic network [3]. These imaging methods were used to infer the localisation of epileptic foci and assist in the design of intracranial electroencephalographic (EEG) recording strategies [4]. These techniques demonstrate the complex relations between normal and abnormal structural and functional data and can be used to direct precise intracranial navigation and surgery for individual patients [4]. 

## Case presentation

**Figure 1:**
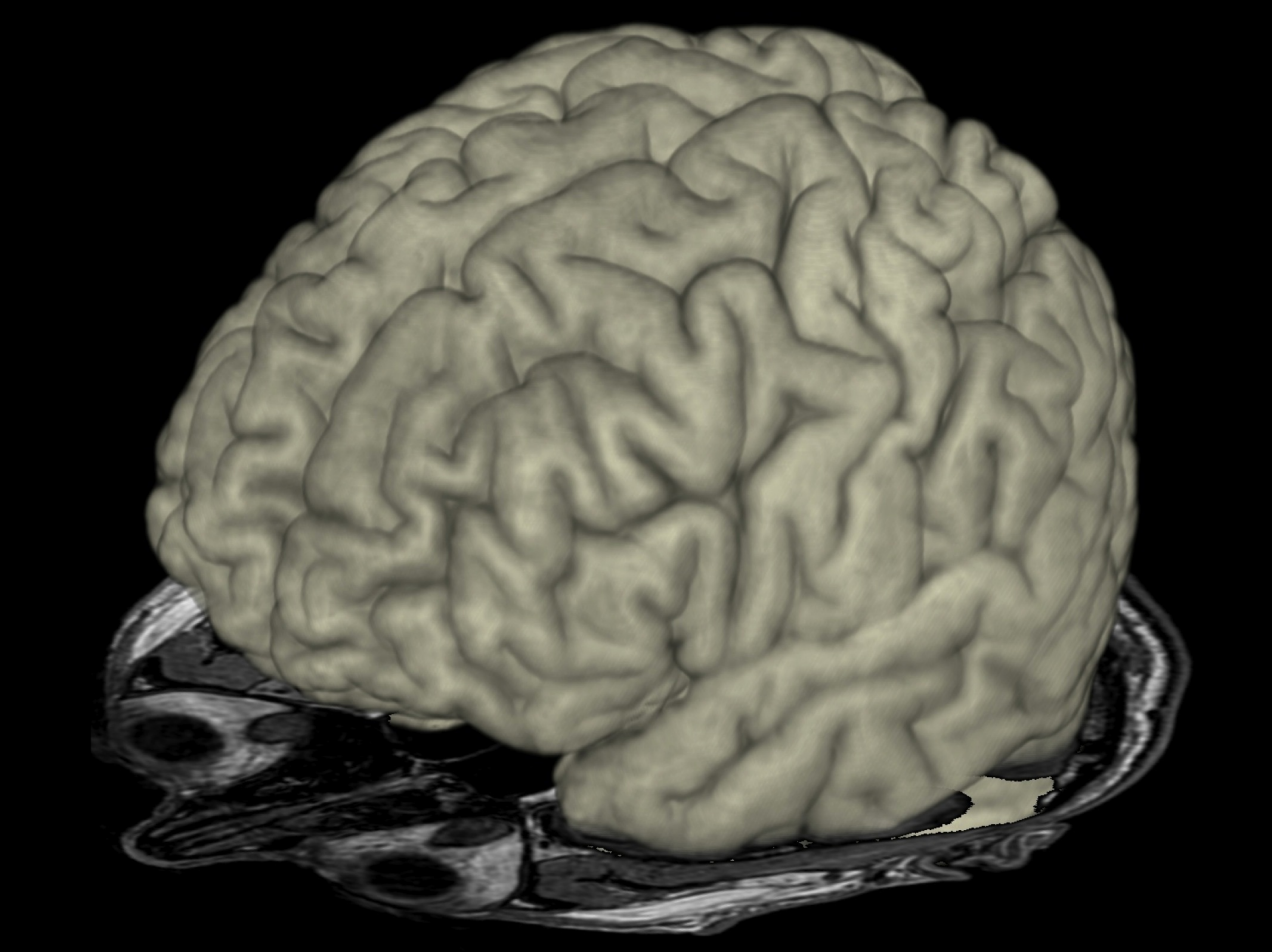
Three-dimensional magnetic resonance imaging (MRI) Three-dimensional MRI of the patient's brain showing no abnormalities

This 41-year-old tailor has had epileptic seizures since the age of 27. He described rare right-sided somatosensory auras and clonic seizures; more commonly, however, he had seizures characterized by complex motor automatisms which at times evolved into hypermotor seizures [5]. The etiology of his epilepsy remained unknown. His conventional high-resolution 3-Tesla MRI was normal (Figure 1). 

**Figure 2:**
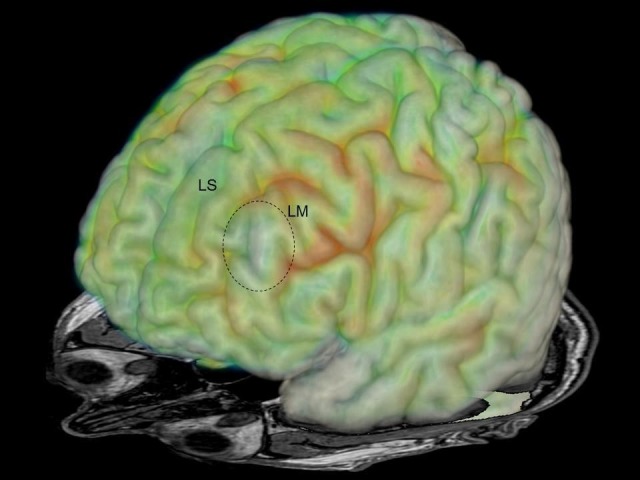
Image fusion of MRI and FDG-PET Image fusion of MRI and FDG-PET demonstrating hypometabolisms in the left medial frontal gyrus (dotted circle). LS: left superior frontal gyrus; LM: left middle frontal gyrus; MRI: magnetic resonance imaging; FDG-PET: fluorodeoxyglucose positron emission tomography

Repeated routine EEGs did not reveal any abnormalities. His neuropsychological evaluation was within normal limits. With medication, generalized convulsive seizure occurred rarely. Most seizures occurred during night sleep. He was resistant to several first and second choice antiepileptic drugs. During non-invasive EEG-video monitoring, right somatosensory auras and hypermotor seizures were recorded, which were consistently associated with left frontal EEG-seizure patterns. No interictal epileptiform discharges were recorded. FDG-PET revealed an area of circumscribed hypometabolism in the anterior part of left medial frontal gyrus (Figure 2). 

**Figure 3:**
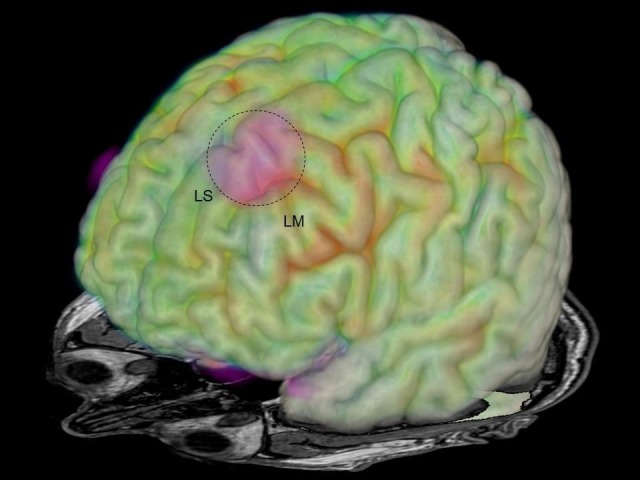
Image fusion of MRI, FDG-PET, and DTI-MRI Image fusion of MRI, FDG-PET, and reduced U-fiber density in the DTI-MRI (marked red & dotted circle) in the left superior frontal gyrus. LS: left superior frontal gyrus; LM: left middle frontal gyrus; MRI: magnetic resonance imaging; FDG-PET: fluorodeoxyglucose positron emission tomography; DTI: diffusion tensor imaging

Diffusion tensor imaging MRI (DTI-MRI) revealed a region of reduced U-fiber density compared to normal controls in the left superior frontal gyrus (Figure 3) [6]. 

**Figure 4:**
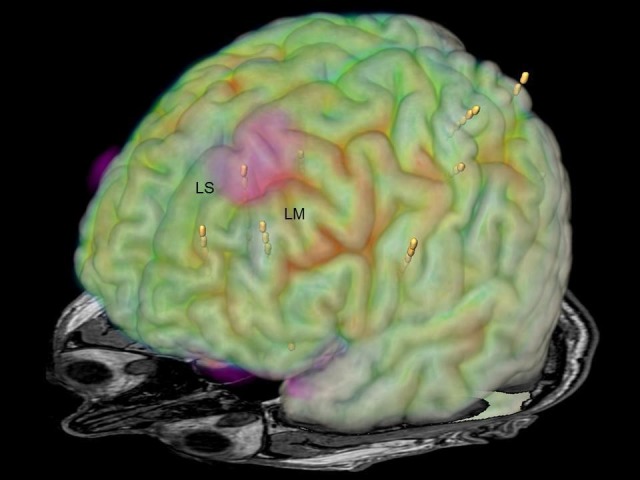
Image fusion of MRI, FDG-PET, DTI-MRI, and depth electrodes Image fusion of MRI, FDG-PET, reduced U-fiber density in DTI-MRI (marked red), and localization of stereotactically implanted depth electrodes (yellow dots). LS: left superior frontal gyrus; LM: left middle frontal gyrus; MRI: magnetic resonance imaging; FDG-PET: fluorodeoxyglucose positron emission tomography; DTI: diffusion tensor imaging

Seven depth electrodes were stereotactically implanted to cover the left frontal, insular, and paracentral cortex (Figure 4). 

**Figure 5:**
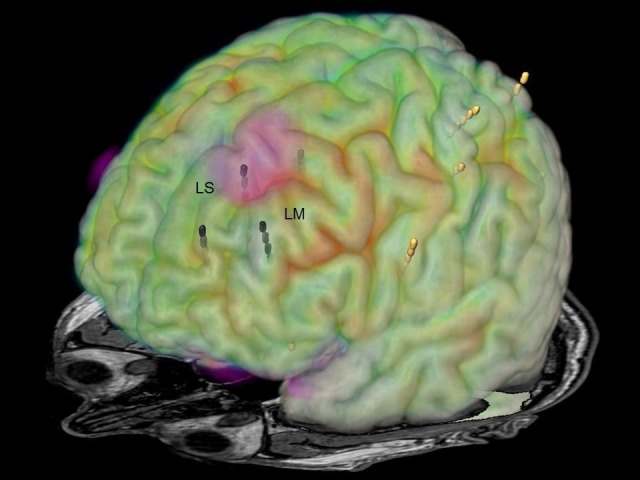
Image fusion of MRI, FDG-PET, DTI-MRI, and localisation of interictal epileptiform discharges Image fusion of MRI, FDG-PET, reduced U-fiber density in DTI-MRI (red marked area in the left superior frontal gyrus) and stereotactically implanted depth electrodes. The depth electrodes showing the seizure onset and the area of maximal interictal epileptiform discharges are marked black. LS: left superior frontal gyrus; LM: left middle frontal gyrus; MRI: magnetic resonance imaging; FDG-PET: fluorodeoxyglucose positron emission tomography; DTI: diffusion tensor imaging

During invasive EEG-video-monitoring, several hypnopompic seizures, complex-motor and hypermotor seizures were recorded which arose from the left middle and the superior frontal gyri (Figure 5; black marked electrodes). 

**Figure 6:**
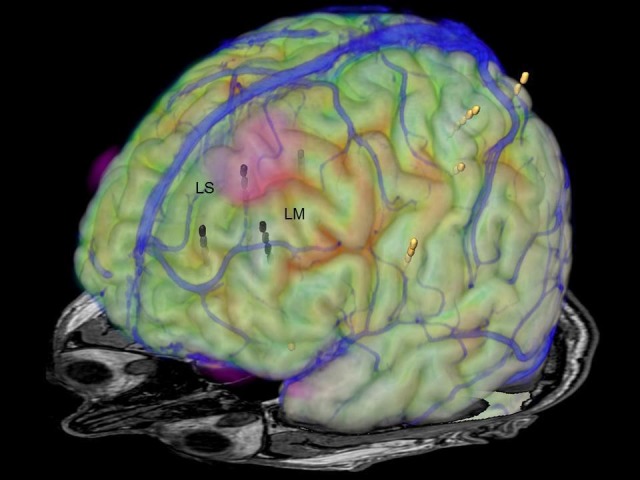
Image fusion of superficial veins, MRI, FDG-PET, DTI-MRI and depth electrodes Superimposition of the superficial veins (blue) on MRI, FDG-PET, DTI-MRI, and the depth electrodes (yellow electrodes). The seizure onset zone and regions of maximal interictal epileptiform discharges are in the electrodes marked black. LS: left superior frontal gyrus; LM: left middle frontal gyrus; MRI: magnetic resonance imaging; FDG-PET: fluorodeoxyglucose positron emission tomography; DTI: diffusion tensor imaging

Coregistration of superficial blood vessels allowed a detailed planning of the resection boundaries (Figure 6). 

**Figure 7:**
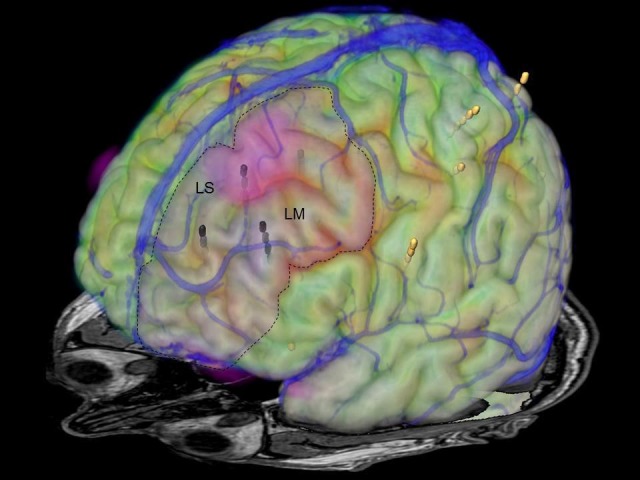
Planned resection The planned resection in the left superior and medial frontal gyri is colored translucent pink (dotted line). LS: left superior frontal gyrus; LM: left middle frontal gyrus

**Figure 8:**
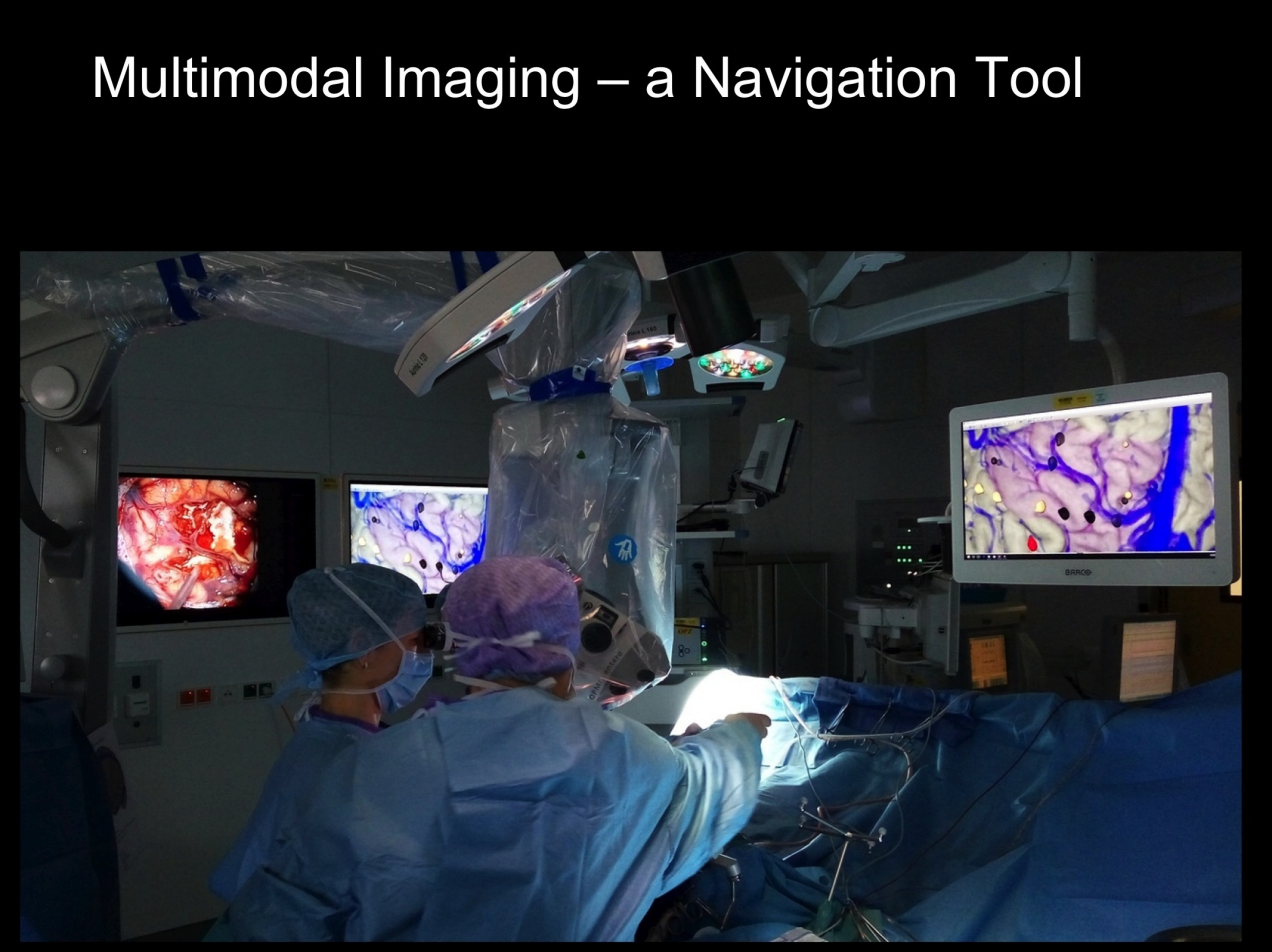
In the operating room The entire image fusion set is available to the neurosurgeon in the operating room.

The resection volume is defined in the multimodal three-dimensional dataset (Figure 7), which is also available intraoperatively during the resection procedure for anatomical reference (Figure 8).

He underwent resection of the middle and superior frontal gyri as shown in Figure 7. His postoperative hospital course was uneventful, and he was discharged a week after the resection. The patient has been seizure-free for nine months with antiepileptic medication.

## Discussion

The role of MRI is crucial for resective epilepsy. Several etiologies, such as cortical malformations, hippocampal sclerosis, or cavernomas, can only be visualized with MRI. Patients in whom conventional MRI does not reveal any abnormality will be more difficult to select for resective surgery and seem to have a poorer outcome regarding seizure freedom [6]. Invasive EEG recordings are mandatory in these patients to identify the epileptogenic zone and delineate eloquent cortex in order to avoid postoperative deficits [3]. Imaging studies, such as functional MRI, provide information on eloquent areas (language, motor function) prior to invasive evaluations and guide the placement of the invasive electrodes. However, invasive evaluation is warranted only if the hypothesized epileptogenic zone is resectable and located outside of the eloquent cortex. 

The different diagnostic tools (EEG, MRI, PET, SPECT, seizure semiology, and neuropsychology) applied to delineate the epileptogenic zone reflect the different aspects of epileptogenicity [3]. It is well established that in extratemporal epilepsies, for instance, the interictal and ictal EEG results in most patients do not co-localize with the MRI documented lesion [7]. The image fusion of the different diagnostic tools, such as MRI, FDG-PET, DTI-MRI, and EEG results, as documented in-depth electrodes and cortical vessels, help to individually plan the resection in view of the epileptogenic zone and neurosurgical requirements of resection [8]. Modern technology allows for a visualization of the three-dimensional fusion image from different angles (Video 1).

**Video 1:** Rotation of three-dimensional image fusion

## Conclusions

In summary, we demonstrate with this case report that image fusion provides valuable information for the surgical procedure in patients considered for individually tailored resective epilepsy surgery, particularly if a conventional MRI is normal.
